# Three-dimensional characterization of fibroblast foci in idiopathic pulmonary fibrosis

**DOI:** 10.1172/jci.insight.86375

**Published:** 2016-04-21

**Authors:** Mark G. Jones, Aurélie Fabre, Philipp Schneider, Francesco Cinetto, Giacomo Sgalla, Mark Mavrogordato, Sanjay Jogai, Aiman Alzetani, Ben G. Marshall, Katherine M.A. O’Reilly, Jane A. Warner, Peter M. Lackie, Donna E. Davies, David M. Hansell, Andrew G. Nicholson, Ian Sinclair, Kevin K. Brown, Luca Richeldi

**Affiliations:** 1 Academic Unit of Clinical and Experimental Sciences, Faculty of Medicine, University of Southampton, Southampton, United Kingdom.; 2 National Institute for Health Research Respiratory Biomedical Research Unit, University Hospital Southampton, Southampton, United Kingdom.; 3 Department of Histopathology, St. Vincent’s University Hospital, Elm Park, Dublin, Ireland.; 4 μ-VIS X-ray Imaging Centre, Faculty of Engineering and the Environment, University of Southampton, Southampton, United Kingdom.; 5 Clinical Immunology, Department of Medicine, Padua University, Padua, Italy.; 6 Department of Cellular Pathology and; 7 Department of Cardiothoracic Surgery, University Hospital Southampton, Southampton, United Kingdom.; 8 Mater Misericordiae University Hospital, Dublin, Ireland.; 9 School of Medicine and Medical Science, University College Dublin, Dublin, Ireland.; 10 Institute for Life Sciences, University of Southampton, Southampton, United Kingdom.; 11 Department of Radiology and; 12 Department of Histopathology, Royal Brompton Hospital and National Heart and Lung Institute, Imperial College, London, United Kingdom.; 13 Department of Medicine, National Jewish Health, Denver, Colorado, USA.

## Abstract

In idiopathic pulmonary fibrosis (IPF), the fibroblast focus is a key histological feature representing active fibroproliferation. On standard 2D pathologic examination, fibroblast foci are considered small, distinct lesions, although they have been proposed to form a highly interconnected reticulum as the leading edge of a “wave” of fibrosis. Here, we characterized fibroblast focus morphology and interrelationships in 3D using an integrated micro-CT and histological methodology. In 3D, fibroblast foci were morphologically complex structures, with large variations in shape and volume (range, 1.3 × 10^4^ to 9.9 × 10^7^ μm^3^). Within each tissue sample numerous multiform foci were present, ranging from a minimum of 0.9 per mm^3^ of lung tissue to a maximum of 11.1 per mm^3^ of lung tissue. Each focus was an independent structure, and no interconnections were observed. Together, our data indicate that in 3D fibroblast foci form a constellation of heterogeneous structures with large variations in shape and volume, suggesting previously unrecognized plasticity. No evidence of interconnectivity was identified, consistent with the concept that foci represent discrete sites of lung injury and repair.

## Abstract 2

Integrated histological and microCT analyses reveal that 3D fibroblast foci are discrete structures with marked variations in shape and volume, suggesting previously unrecognized plasticity.

## Introduction

Idiopathic pulmonary fibrosis (IPF) is the prototypic fibrotic lung disease in which progressive scarring of the lungs leads to death by respiratory failure (1). The median survival of 3 years from diagnosis is worse than many cancers (2). Despite identification of activated fibroblasts as key effector cells pathologically remodeling the extracellular matrix, therapeutic options remain limited, and a better understanding of disease pathogenesis is required (3–5).

Diagnosis of IPF is based on recognition of the usual interstitial pneumonia (UIP) pattern on chest high-resolution CT or surgical lung biopsy (1). The histological pattern of UIP is believed to be produced by the consequences of repeated epithelial injury together with unfettered fibroproliferation (6). Aggregates of proliferating fibroblasts and myofibroblasts, termed “fibroblast foci,” are a key histological diagnostic feature of UIP thought to represent areas of active fibrosis (7). On standard 2D pathologic examination fibroblast foci are considered small, distinct lesions, and their profusion has been reported to be of prognostic significance, although study findings vary (8–12). They have been proposed to be linked in a complex reticulum that is highly interconnected and extends from the pleura into the underlying parenchyma (13), yet the 3D morphology and spatial interrelationships of fibroblast foci remain poorly understood.

Micro-CT allows nondestructive imaging of tissue 3D microarchitecture, down to spatial resolutions, in the order of 1 to 10 μm (14, 15). It has informed our understanding of distal airways disease in patients with end-stage chronic obstructive pulmonary disease and chronic lung allograft rejection (16, 17). In fibrotic lung diseases, micro-CT imaging has been employed for quantification of fibrosis in small animal studies (18–23); however, as samples have required processing with contrast agents or air inflation prior to fixation, application to diagnostic human interstitial lung disease (ILD) tissue samples has not been possible, with extremely limited tissue access. We have recently developed a protocol that allows imaging of routinely prepared formalin-fixed paraffin-embedded human lung tissue samples by micro-CT (24). This nondestructive process can be applied to tissue used for diagnosis, and conventional histopathologic sectioning of the tissue can subsequently be performed; this enables cellular or molecular information from methodologies such as immunohistochemistry or in situ hybridization to be precisely coregistered within the volumetric data set from micro-CT.

Given the pivotal nature of fibroproliferation to the progression of IPF and the key role that fibroblast foci are believed to play in this process (6), new insights into the structure of these lesions may further advance our understanding of whether they are interlinked in a complex reticulum as the leading edge of a “wave” of fibrosis or they comprise multiple distinct structures that have arisen as a consequence of multifocal lung injury (6, 13). In our study, we applied an integrated micro-CT and histological methodology on formalin-fixed paraffin-embedded diagnostic lung tissue to study the morphology of fibroblast foci in 3D. Our work demonstrates the potential for 3D imaging methodologies to further inform concepts of the underlying pathogenesis of fibrotic lung diseases and reveals that, in contrast to current understanding from standard pathology of 2D tissue sections, in 3D fibroblast foci form a constellation of heterogeneous structures, with large variations in shape and volume, suggesting previously unrecognized plasticity. We identify no evidence of general interconnectivity, consistent with the concept that foci represent discrete sites of lung injury and repair.

## Results

### Micro-CT of formalin-fixed paraffin-embedded lung tissue identifies diagnostic features of the UIP pattern.

**Figure 1:**
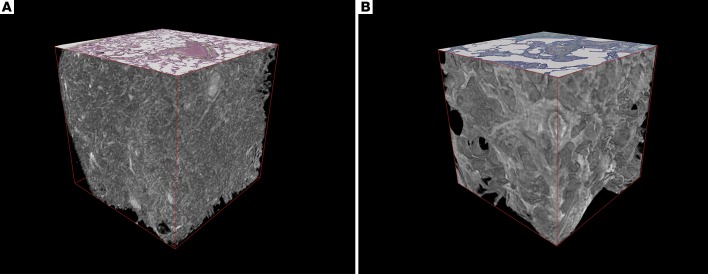
3D volume renderings of normal and usual interstitial pneumonia/idiopathic pulmonary fibrosis lung tissue imaged by micro-CT. Cubes (2 mm per side) of (**A**) normal lung tissue and (**B**) usual interstitial pneumonia (UIP)/idiopathic pulmonary fibrosis (IPF) lung tissue were digitally extracted from the reconstructed micro-CT lung tissue volumes following removal of paraffin by absolute thresholding. For reference on the superior surface, a coregistered histological section stained with Movat’s Pentachrome is displayed. Morphological differences in 3D are apparent with UIP/IPF tissue, demonstrating loss of alveolar structures and increased parenchymal thickness, with an impression of “fullness” within the UIP/IPF tissue.

**Figure 2:**
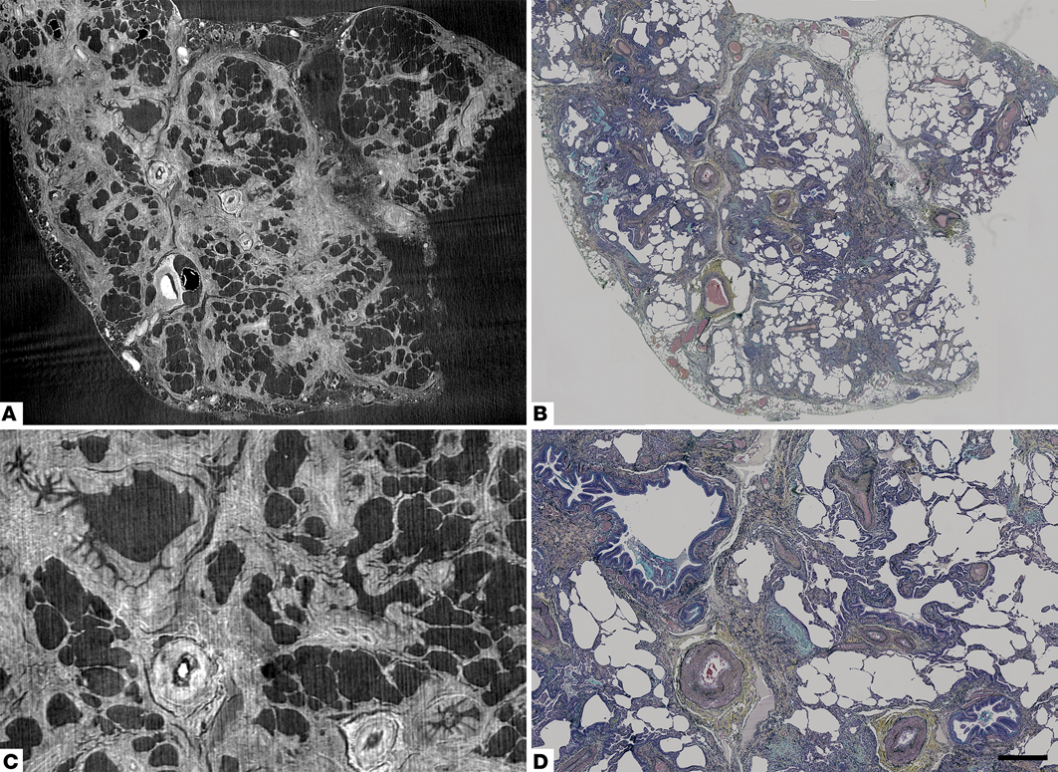
Correspondence of structural details identified by micro-CT to those seen by light microscopy. (**A** and **C**) Registration-based image matching of a histological section of usual interstitial pneumonia/idiopathic pulmonary fibrosis tissue stained with Movat’s Pentachrome and (**B** and **D**) corresponding micro-CT images. Images in **C** and **D** are subareas of **A** and **B**. Scale bar: 1,400 μm (**A** and **B**); 500 μm (**C** and **D**).

First, we assessed visualization of diagnostic paraffin-embedded lung tissue 3D microarchitecture by micro-CT. Micro-CT enabled interactive multiplanar assessment of tissue microarchitecture (Supplemental Video 1; supplemental material available online with this article; doi:10.1172/jci.insight.86375DS1). 3D reconstructions identified clear morphological differences between normal and IPF lung tissue, with volume renderings in Figure 1 demonstrating loss of alveolar structures and increased parenchymal thickness in IPF, consistent with previous stereological findings (25). Supplemental Videos 2 and 3 visualize rotation of these volume renderings. Feature correspondence between corresponding histological sections and micro-CT slices of IPF lung tissue was assessed, with qualitative comparison (Figure 2) demonstrating that micro-CT can visualize normal lung structural features, such as airways, blood vessels, and alveolar airspaces, in addition to structural features of the UIP pattern, including heterogeneity of the distribution of interstitial fibrosis and normal appearing lung, architectural distortion, and honeycomb change.

### Fibroblast foci are locally complex, heterogeneous structures in 3D.

**Figure 3:**
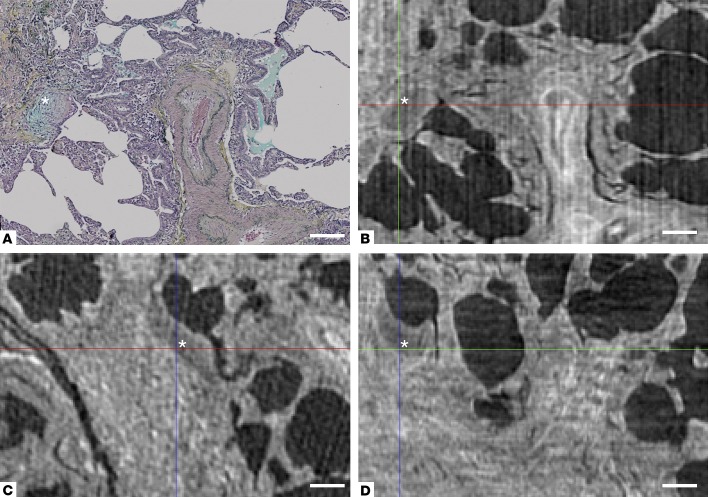
Multiplane visualization of a fibroblast focus with micro-CT. (**A**) Movat’s Pentachrome stain of usual interstitial pneumonia/idiopathic pulmonary fibrosis tissue, with a fibroblast focus identified by the asterisk. The corresponding area of the micro-CT volume is seen in the *xy* plane in **B**. In addition, the focus can also be visualized (**C**) in the *xz* plane and (**D**) in the *yz* plane, enabling further assessment of the 3D morphology of fibroblast foci. Scale bar: 200 μm.

We then studied whether fibroblast foci were identifiable in micro-CT image data. Correlation of histological slices and coregistered micro-CT images established features that typically enabled identification of fibroblast foci greater than 200 μm when integrated with visualization in 3 different planes (*xy*, *xz*, *yz*), specifically when the foci were adjacent to airspaces, given their reduced gray scale intensity when compared with surrounding tissue, as illustrated in Figure 3 and Supplemental Figure 1.

**Figure 4:**
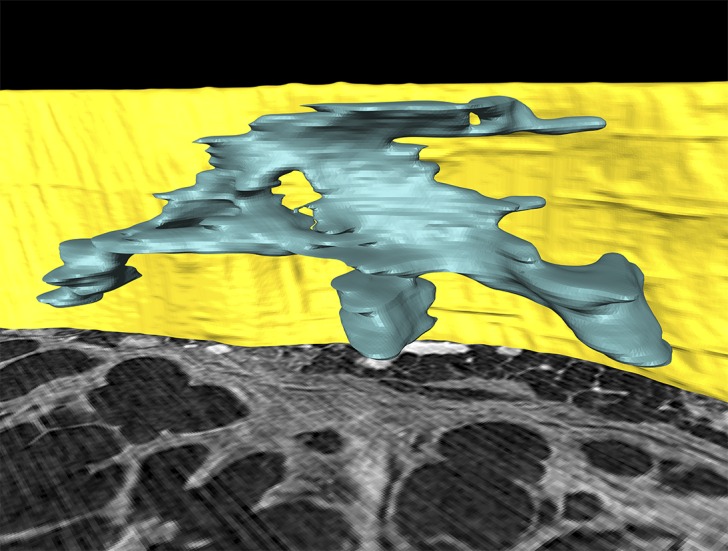
Fibroblast foci are locally complex structures. A computer-generated 3D surface view rendering of one fibroblast focus from micro-CT segmentation identifies that fibroblast foci are locally complex structures in 3D. The focus is blue, and for reference the pleural surface is visualized in yellow (height 1 mm) with a micro-CT slice shown inferiorly.

Initial visual 3-plane micro-CT analyses suggested a locally complex fibroblast focus morphology in 3D that was not apparent on cross-sectional 2D histopathologic examination. To confirm this finding, we performed histological identification of fibroblast foci, followed by segmentation of the corresponding image sections within the micro-CT volume to enable accurate integrated assessment of their 3D structure. Fibroblast foci were digitally labeled in the corresponding micro-CT slice every 8 μm over 1,000 μm, using the segmentation editor, having been identified in the histological sections as aggregates of spindle-shaped cells with a characteristic blue-green color on Movat’s pentachrome–stained sections or pale color on hematoxylin and eosin–stained sections; these stainings represent focal deposits of young, immature collagen and proteoglycans within the subepithelial interstitium. All foci within the micro-CT field of view were labeled. 3D visualization and quantification of the segmented data identified complex discrete structures with marked variation in size and shape (Figure 4, Table 1, Supplemental Figure 2, and Supplemental Video 4) that appear to be a nonuniform process. The 3D render visualized in Figure 4 is available for download (Supplemental Render 1).

**Figure 5:**
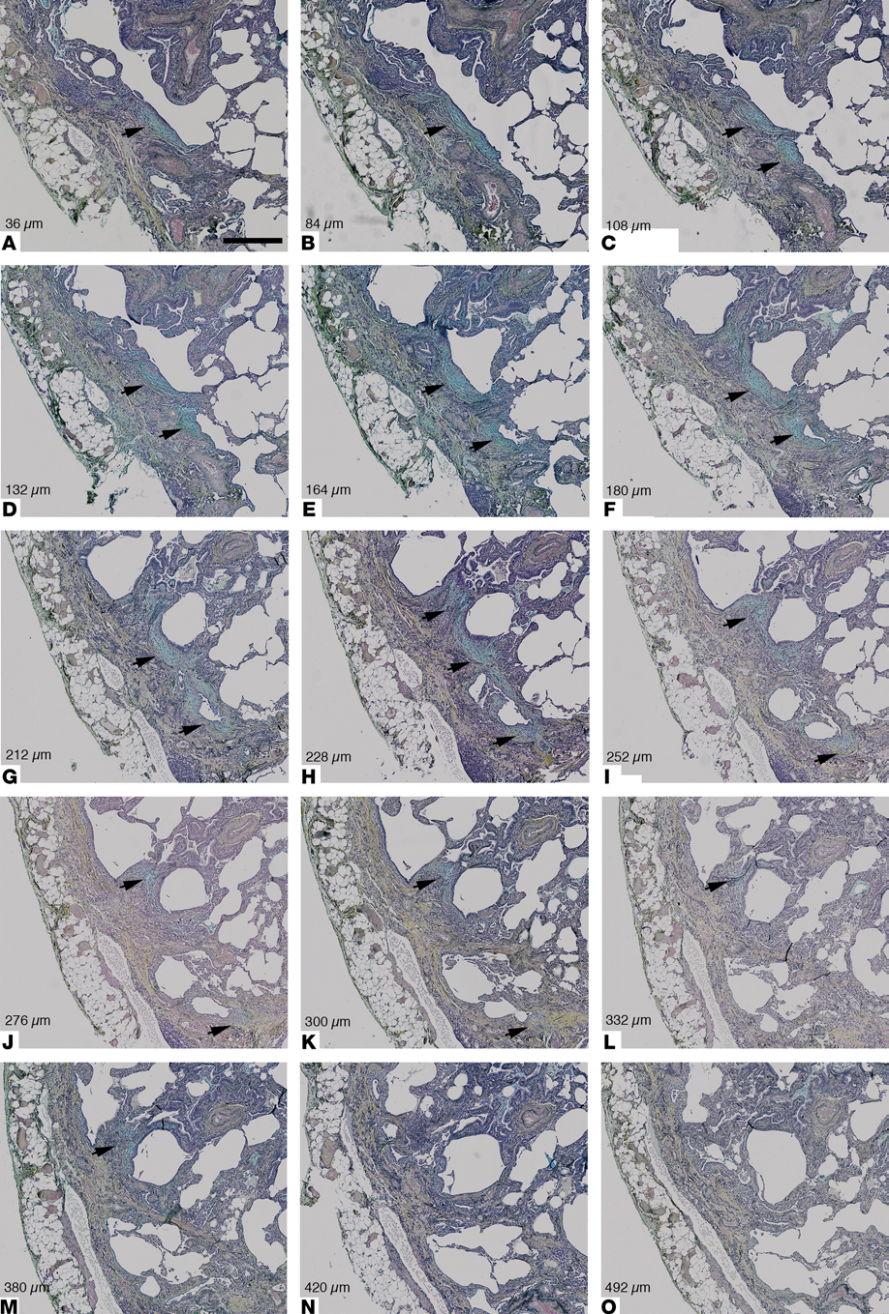
Review of coaligned Movat’s pentachrome–stained sections of an area of usual interstitial pneumonia tissue demonstrates the complex morphology of one fibroblast focus in 3D. Following identification of one fibroblast focus (**A**, black arrow), (**A**–**M**) illustrative photomicrographs demonstrate the changes in morphology of one fibroblast structure when sequential sections are visually reviewed, with apparently discrete foci forming part of one locally complex individual structure in 3D (indicated by black arrows in **B**–**M**). In **N** and **O**, the focus is no longer visible. Section depth in microns is indicated for each photomicrograph. Scale bar: 500 μm. The 3D rendering of this fibroblast focus is visualized in Figure 4 and Supplemental Video 4.

Fibroblast focus volumes ranged from 1.6 × 10^4^ μm^3^ to 9.9 × 10^7^ μm^3^. No evidence of substantial extension of individual foci inwards from the pleura to the parenchyma was identified. Review of digitally imaged sequential 2D histology sections identified the complex morphology of an individual fibroblast focus in 3D, with a number of apparently independent fibroblast foci in 2D identified to form one morphologically discrete structure. These foci ranged from relatively simple 3D structures (Supplemental Figure 3) to more morphologically complex structures adjacent to established fibrosis (Figure 5).

### Fibroblast foci are not generally interconnected.

**Figure 6:**
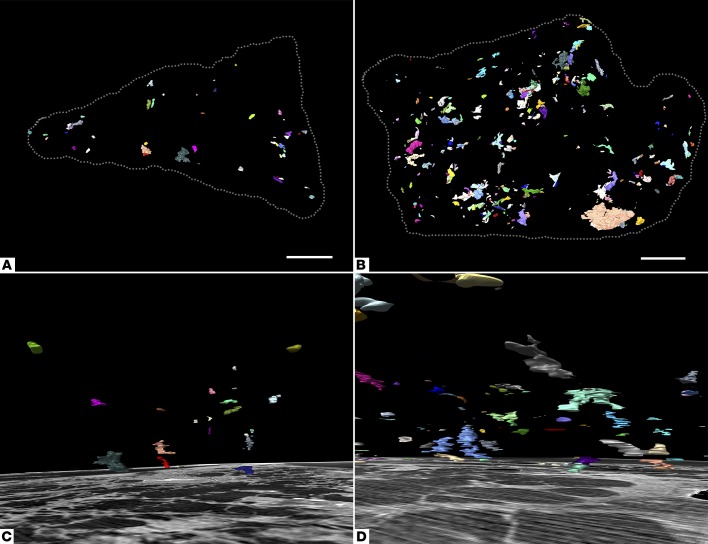
Fibroblast foci are discrete structures in 3D. Computer-generated 3D surface view renderings of all fibroblast foci from two micro-CT segmentations. (**A** and **C**) Case 2 has the lowest number density of fibroblast foci per mm^3^ of lung tissue. (**B** and **D**) Case 3 has the highest number density. In **A** and **B**, a 3D surface view rendering is shown in the *xy* plane (corresponding to the histological sectioning plane) with tissue boundaries marked by the dashed gray lines. In **C** and **D**, a 3D surface view rendering is shown with a corresponding micro-CT slice displayed inferiorly (depth 1 mm). Scale bar: 2 mm.

Micro-CT 3-plane visualization, 3D segmentations (Figure 6 and Supplemental Video 5), and sequential 2D histology section review were evaluated for evidence of fibroblast focus interconnectivity at a larger scale, with numerous independent multiform fibroblast structures identified in each tissue volume and fibroblast focus frequency ranging from 0.9 to 11.1 per mm^3^ of lung tissue. The Euler number, a measure of redundancy in a network, was calculated for each case and identified no evidence of fibroblast focus interconnectivity (see Supplemental Table 1 and refs. 26, 27).

Given the identified variability of foci in 3D, fibroblast focus profusion on 2D histological sections was then semiquantitatively and quantitatively assessed over a depth of 1 mm of lung tissue from each patient. Analyses identified variability both quantitatively (up to 4-fold intratissue variation) and semiquantitatively (Supplemental Figure 4). An exploratory analysis investigating the association between 3D fibroblast focus density and change in forced vital capacity (FVC) over 1 year identified a negative correlation (Supplemental Figure 5).

## Discussion

Through an integrated histological and micro-CT analysis of IPF lung tissue we characterized fibroblast foci in 3D, identifying that they are morphologically complex structures with large variations in shape and volume. Within each tissue sample, numerous discrete fibroblast structures were present, without evidence of general interconnectivity.

While IPF remains an idiopathic disease, there have been several proposals regarding mechanisms of pathogenesis, one of which is that fibroblast foci form a highly complex and interconnected reticulum extending from the pleura to the lung parenchyma as a “wave” of progressive fibroproliferation (13, 28, 29). This concept was informed by a study from Cool et al., in which a computer-generated 3D reconstruction and Euler connectivity analysis of histological sections over a depth of 500 μm were proposed to demonstrate an interconnected fibroblast reticulum (13). However, the methodological advances of our work enabled analysis of far larger tissue volumes than previously possible, and we identified that the preceding reconstruction scale was consistent with visualization of one fibroblast focus of comparable dimensions to that shown in Figure 4. With multiple methodologies, our analyses showed that fibroblast foci are independent, discrete structures and, as a group, are not part of a reticulum. In support of this finding, human androgen receptor gene methylation assay analysis of foci by Cool et al. identified balanced methylation, indicating polyclonality of foci (13). Our results are consistent with the concept that fibroblast foci represent discrete nonconnected sites of localized lung injury, with 3D reconstructions identifying a constellation of independent fibroblast structures in support of the paradigm that IPF is a consequence of aberrant wound healing responses to local microinjuries (6).

At a 2D cross-sectional histological level, fibroblast foci are usually considered small, distinct lesions (7, 30), although they are often found grouped in proximity, forming what are thought to be areas of active fibrosis. Our results showed that, in 3D, foci in proximity and typically with shared areas of established fibrosis form one complex discrete fibroblast structure. A heterogeneous population was identified with large variations in size and shape and over a 100-fold difference between largest and smallest volume fibroblast foci. Acknowledging that the cause of lung injury in IPF has yet to be determined, we speculated a number of possibilities for this finding, which suggests previously unrecognized plasticity. It is possible that the size and morphology of an individual focus reflects the extent and distribution of injury at a lung surface. Alternatively, volume variation could indicate temporal growth of foci, with the smallest foci occurring at the most recent sites of injury. The latter possibility is in keeping with our identification of small isolated foci. Further work would be required to investigate these relationships in more detail.

While increased profusion of fibroblast foci has been proposed to correlate with mortality in patients with IPF, a number of studies have identified no association, and the clinical significance of this finding remains uncertain (8–12). Differences in patient selection and methodology have been suggested to account for discordant findings, with marked variations in methodologies, including semiquantitative scoring, quantitative point counting of random fields, or whole-slide fibroblast focus quantification. To our knowledge, no previous study has systematically assessed variability of foci within tissue samples. Our data indicated that fibroblast focus profusion can vary markedly over a 1-mm depth of tissue; however, studies correlating profusion with mortality have typically reviewed one or two tissue sections per lobe sampled. Focus variability within tissue samples should therefore be considered in future studies correlating fibroblast focus profusion with clinical outcome. In support of the concept of fibroblast foci as a marker of disease activity was our finding of a possible association between 3D fibroblast focus density and FVC change. However, given our limited sample size larger studies would be required to confirm such an association.

Although significant progress has been made, our understanding of the pathogenesis and treatment of IPF remains limited (6). Nintedanib and pirfenidone are two novel antifibrotic drugs that were recently shown to approximately halve the rate of decline of lung function in IPF (4, 5), with both having multiple actions on the proliferation and signaling pathways of fibroblasts and myofibroblasts (31–34). Additional therapies are urgently required since these two drugs only reduce the rate of disease progression. This study emphasizes the importance of investigating new agents which prevent and/or ameliorate the repeated lung injuries that result in multifocal sites of fibroproliferation.

Initial work correlated micro-CT of whole paraffin-embedded samples of UIP tissue with histological sections. While soft tissue visualization through conventional X-ray–based CT is a challenge due to the low X-ray absorption of soft tissues, our micro-CT imaging protocol was optimized to provide the necessary image contrast between soft tissue and paraffin and also between different microarchitectural features within the tissue itself (24). Excellent microarchitectural feature correlation between histology and micro-CT imaging was observed, and diagnostic structural features of a pattern of UIP were visualized. Whereas larger fibroblast foci were directly visible within micro-CT image data, typically those less than 200 μm were not. In the future, identification and segmentation of smaller fibroblast foci may be possible through improved contrast resolution, thresholding, and pattern recognition approaches. Automated approaches, based on exploiting the X-ray attenuation contrast window between tissue and paraffin mounting medium as well as between different tissue compartments may further facilitate subsequent visualization and quantification.

There are significant advantages to the use of paraffin-embedded tissue to study fibrotic ILDs. While images of paraffin-embedded tissue have a lower contrast than air-inflated, fixed, and dried tissue samples, access to sufficient fresh or frozen diagnostic tissue with robust histopathological integration poses significant challenges in fibrotic ILDs. The availability of large banks of archived diagnostic paraffin-embedded samples facilitates systematic micro-CT analyses of fibrotic ILDs. Similarities and differences may further advance our understanding of underlying pathophysiology and potentially inform stratification approaches in fibrotic lung diseases. Given the diagnostic challenges that ILD histopathology poses, the ability to visualize in 3D could ultimately have diagnostic application. Furthermore, use of paraffin-embedded samples allows integration of coregistered histology sections within the 3D micro-CT volumetric data set; this enables accurate 3D analyses of the relationship of cellular and microarchitectural features of fibrotic lung diseases. While a predominantly manual segmentation methodology was applied during this initial study, development of automatic and semiautomatic methodologies will further enable quantitative feature extraction and analyses such as 3D topographical distribution of lung fibrosis.

Our study has demonstrated that the application of 3D imaging methodologies can further advance concepts of disease pathogenesis in fibrotic lung diseases. It has indicated that fibroblast foci are heterogeneous and of varying size, complexity, and frequency in each patient’s biopsy, suggesting previously unrecognized plasticity. We have also shown that these fibroblast structures are independent of each other, consistent with their being the product of discrete sites of lung injury and repair.

## Methods

### Clinical material.

Clinically indicated diagnostic surgical lung biopsy specimens from 4 patients (see Table 2 for further clinical details) with a subsequent multidisciplinary diagnosis of IPF according to international consensus guidelines were studied (1). No patient had clinical or radiological evidence of an acute exacerbation prior to or at the time of biopsy. Specimens had been diagnosed as showing a typical UIP pattern, confirmed by the independent review of two expert pulmonary pathologists. The samples had received standard processing with fixation in neutral buffered formalin for 48 hours and embedding in paraffin wax. Control formalin-fixed paraffin-embedded healthy lung tissue was from macroscopically normal lung sampled remote from the cancer site in two patients undergoing surgery for lung cancer.

### Micro-CT imaging protocol.

The paraffin-embedded lung samples were scanned using a custom-built Nikon Metrology micro-CT scanner at an isotropic voxel size of 8 μm as previously described (24), with optimization to maximize soft tissue contrast. An X-ray tube potential peak of 55 kVp was used at a beam current of 104 to 114 μA. 2,601 tomographic radiographic viewpoints of the samples were assessed (360° rotation in 0.14° steps) by acquiring 64 repeated projections (2,000 × 2,000 pixels) for each angular step to increase signal-to-noise ratio through frame averaging, where integration time for individual projections was set to 500 ms at an isotropic voxel size of 8 μm, resulting in a field of view of 16 × 16 mm^2^. Together with sample shuttling during acquisition to suppress ring artefacts with the reconstruction, the gross image acquisition time per sample was about 24 hours. The projections were then reconstructed in 3D using the Feldkamp, Davis, and Kress algorithm for cone beam tomography in the DigiR3D tomography reconstruction module of the DigiXCT software suite (Digisens) or using standard filtered-back projection within CTPro3D (v. XT 2.2 service pack 10, Nikon Metrology) and CTAgent (v. XT 2.2 service pack 10, Nikon Metrology) (35).

### Histology acquisition.

Following micro-CT imaging 4-μm-thick serial sections were cut over an approximate depth of 1,000 μm. Sections every 8 μm (corresponding to the actual micro-CT voxel size of 8 μm) were deparaffinized and stained using modified Movat’s pentachrome or hematoxylin and eosin stain (36). Whole sections were imaged using a ×20 objective on a Dot-Slide scanning system (Olympus) and visualized with Olyvia 2.6 (Olympus).

### Micro-CT image visualization and correlation with histological sections.

Image preprocessing, segmentation, 3D volume reconstruction, and quantification of lung sample micro-CT data were performed with AvizoFire 7.1 (FEI). Three or more structural features in each micro-CT volume were matched to a specific histological section, and the 3D orientation of the micro-CT volume was correspondingly transformed for plane correspondence between histological and micro-CT data. For direct comparisons of histological and micro-CT sections, the Fiji distribution of the ImageJ (version 1.49p) plugin Landmark Correspondences was used to coregister the images after the initial matching for plane correspondence (37, 38).

### Micro-CT segmentation and analyses.

Fibroblast foci were digitally labeled in the corresponding micro-CT slice every 8 μm over 1,000 μm, using the segmentation editor, having been identified in the histological sections as aggregates of spindle-shaped cells by a characteristic blue-green color from Movat’s pentachrome stain or a pale color from hematoxylin and eosin stain; these stainings represent focal deposits of young, immature collagen and proteoglycans within the subepithelial interstitium. The contours of fibroblast foci within corresponding micro-CT slices were drawn using the “Lasso” function in AvizoFire. All foci within the micro-CT field of view were labeled. Missing levels (for example due to ribbon section breaks) were manually interpolated. Following tissue segmentation individual foci were then tagged by the labeling function, and individual foci were considered as separate structures if they had no common voxel faces, edges, or corners. The foci were then visualized through volume rendering using a cyclic color map so that foci in close proximity were more likely to be shown in a different color. Volume, 3D shape, and occurrence were quantified. For 3D volume renderings and calculation of lung tissue volume, paraffin was segmented from each micro-CT volumetric data set. Global thresholding was applied, followed by application of the “Smooth Labels” function of AvizoFire, with the threshold identified as the mean gray scale value of a 1-mm^3^ volume of paraffin. An example of these steps is shown in Supplemental Figure 6. Using the “Crop” function of AvizoFire, the subvolume corresponding to the histological stack was then extracted, and lung tissue volume quantified.

### Fibroblast focus quantitation and connectivity analyses.

Connectivity between fibroblast foci was assessed visually through micro-CT 3-plane visualization, 3D foci segmentations, and corresponding sequential 2D histology section review. The total number of foci per case and fibroblast foci tissue density were calculated. Stereological analysis was performed, and fibroblast foci every 20 to 30 μm over approximately 1,000 μm were marked on photomicrographed whole-tissue sections using Olyvia. Fibroblast focus profusion was semiquantitatively (scale 0–6) or quantitatively (fibroblast foci per cm^2^ of tissue) measured on these sections. The Euler-Poincaré characteristic or Euler number (27, 39), a measure of redundancy in a network, was calculated. Full details are provided in the Supplemental Methods.

### Statistics.

All graphs were created using GraphPad Prism software, and statistical analyses were calculated using GraphPad Prism (version 6; GraphPad Software). The correlation between 1 year FVC percentage change and fibroblast focus density was determined by linear regression. A *P* value of less than 0.05 was considered significant.

### Study approval.

The study was approved by the Mid and South Buckinghamshire Local Research Ethics Committee (ref 07/H0607/73), and all subjects gave written informed consent.

## Author contributions

MGJ, AF, PS, DED, and LR conceived the project, designed experiments, analyzed data, and wrote the manuscript. PML, JAW, KMAO, and IS contributed to the project conception, planning, and experimental design. MGJ, AF, GS, FC, MM, and SJ performed experiments and analyzed data. AA, BGM, and KMAO contributed to clinical sample collection. KKB contributed to data interpretation, data review, and manuscript revision. DMH and AGN contributed to the conception and design of the study and reviewed data. All authors reviewed, revised, and approved the manuscript for submission.

## Supplementary Material

Supplemental data

Supplemental Video 1

Supplemental Video 2

Supplemental Video 3

Supplemental Video 4

Supplemental Video 5

Supplemental Render 1

## Figures and Tables

**Table 2:**
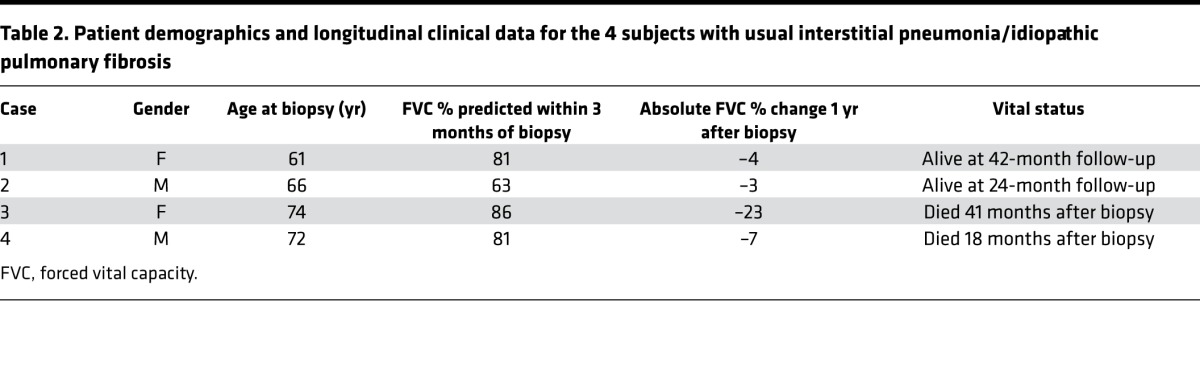
Patient demographics and longitudinal clinical data for the 4 subjects with usual interstitial pneumonia/idiopathic pulmonary fibrosis

**Table 1:**
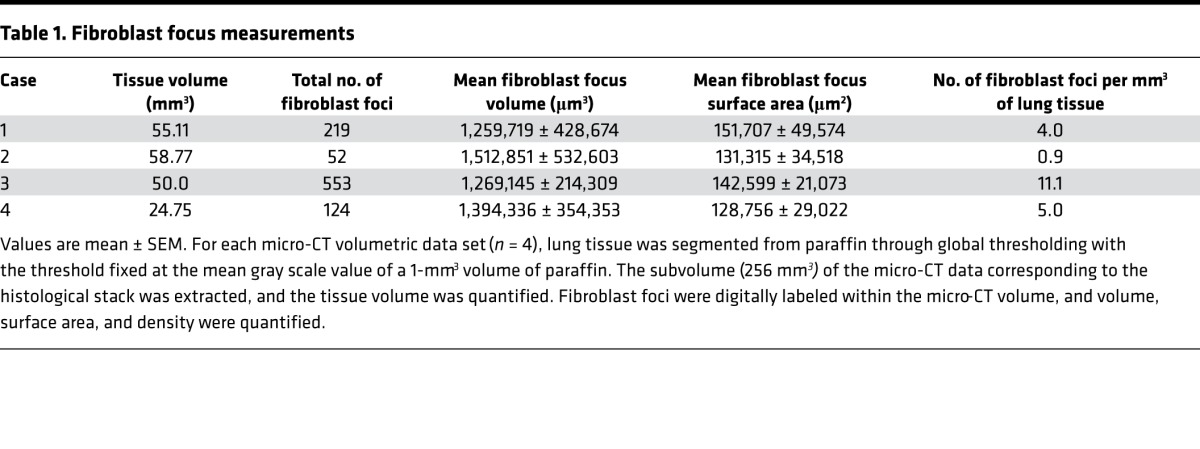
Fibroblast focus measurements
