# Supplementary material for: Three-dimensional characterization of fibroblast foci in idiopathic pulmonary fibrosis
Source: JCI Insight. 2016 Apr 21;1(5):e86375. doi: 10.1172/jci.insight.86375 (PMC4889020; doi:10.1172/jci.insight.86375)
Supplement: Supplemental data [file jciinsight-1-86375-s001.pdf]

Three-Dimensional Characterisation of Fibroblast Foci in Idiopathic Pulmonary Fibrosis

Supplementary Appendix

Supplementary Methods

Fibroblast focus connectivity analysis

Fibroblast foci every 20-30 µm over approximately 1000 µm were marked on photo-micrographed whole-tissue sections using Olyvia 2·6 (Olympus, Southend-on-Sea, UK). Connectivity of fibroblast foci was then assessed by stereological analysis with calculation of the Euler-Poincaré characteristic or Euler number χ_3, a measure of the redundancy of connectivity in a network (S1-6). Using adjacent sections as physical dissectors for counting events in both directions (using each single section once as a sampling section and once as a look-up section) isolated fibroblast foci without connections to previously visible foci were denoted as islands (I) or disconnected parts (tangents with a convex surface), fibroblast foci that spanned sections were designated as bridges (B) or redundant connections (tangents with saddle surface), and foci surrounded entirely by tissue were referred to as holes (H) or internal cavities (tangents with concave surface). The observed dissectors contributed to the Euler number according to:

$$\Delta\chi_3 = \sum I - \sum B + \sum H$$

The greater the number of bridges and holes is compared to islands, the greater the redundancy and the more negative the Euler number becomes. This means that large negative Euler numbers are characteristic for highly connected networks, while a positive number (number of islands and holes greater than the number of bridges) indicates that the fibroblast foci are isolated or unconnected.

Fibroblast focus profusion analyses

Profusion of fibroblast foci every 20-30 µm over approximately 1000 µm was measured. For semi-quantitative analysis the absence of fibroblastic foci was scored as 0, and the most profuse score was 6 after the method described by Nicholson *et al* (S7). For quantitative analysis whole lung tissue area was calculated using the image analysis software cellSens 1.4 (Olympus Southend-on-Sea, UK), and total number of fibroblast foci obtained by counting all foci from marked photo-micrographed whole tissue sections. Fibroblast foci / cm^2 of tissue was then calculated.

Supplementary Tables

Table S1. Stereoscopic analysis of 4 subjects with UIP/IPF and Euler Number (χ_3) calculations and the tissue depth studied. In a connected network the Euler number is a measure of the redundancy of a system, with the more the negative the number the greater the connectivity, and a positive number indicating that fibroblast foci are unconnected.

	**Case 1**	**Case 2**	**Case 3**	**Case 4**
**Depth studied**	964 μm	984 μm	984 μm	984 μm
**Euler number (χ_3)**	8	104	6	12

Supplementary Figures

Figure S1. Visualisation of a fibroblast focus by microCT and histology. Registration based image matching of a histological section of usual interstitial pneumonia tissue stained with Movat's Pentachrome-stain (A) and the corresponding microCT image (B). A fibroblast focus is identified by the asterisk (*). In (C) a 3D volume rendering of the corresponding microCT volume (1 mm per side) identifies the focus. A co-registered histology section is visualised on the superior surface. Scale bar in (A) is 200 µm.

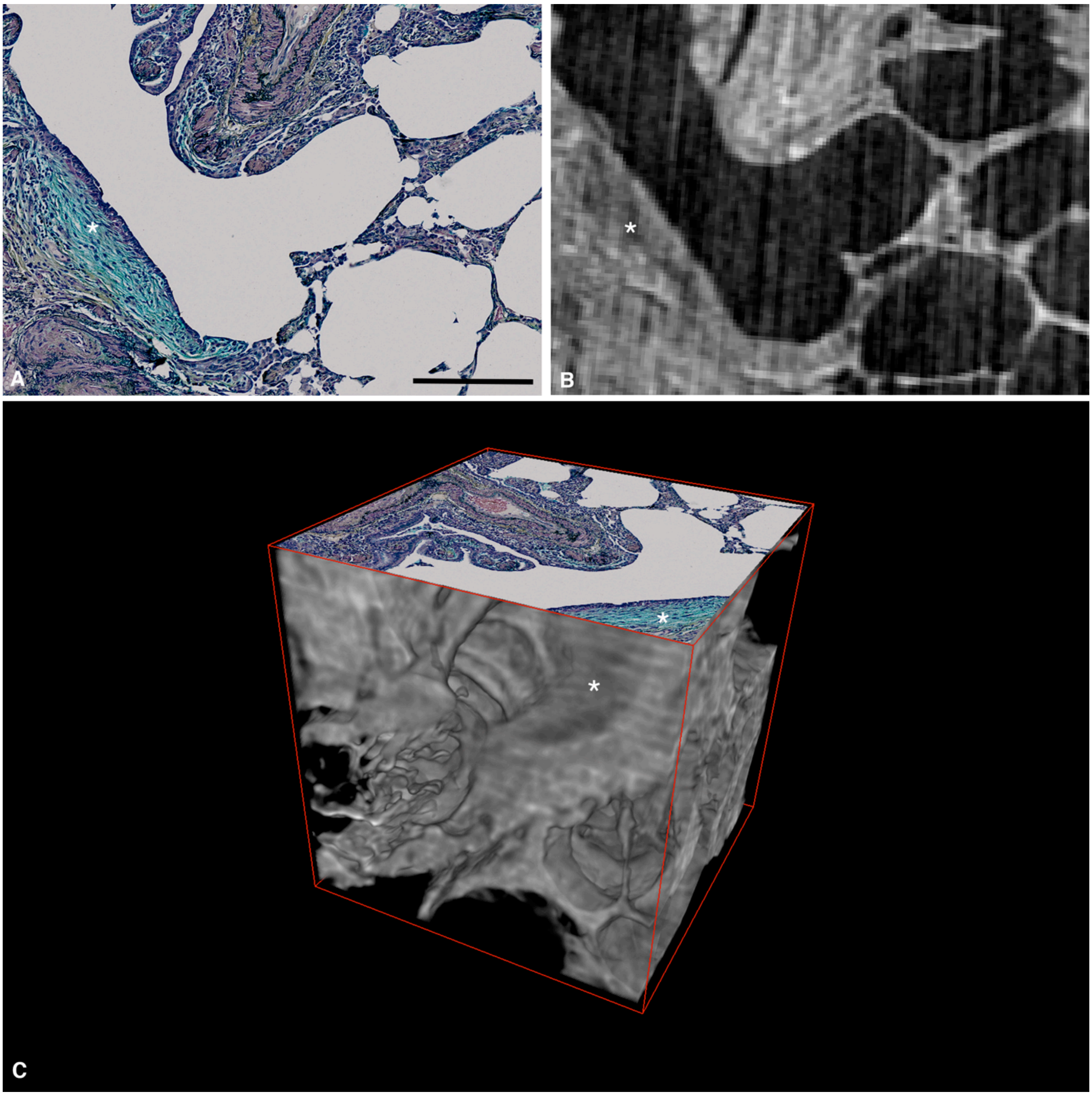

Figure S2. Fibroblast focus volume distribution by case. Fibroblast foci were digitally labelled within the microCT volume, and volumes quantified.

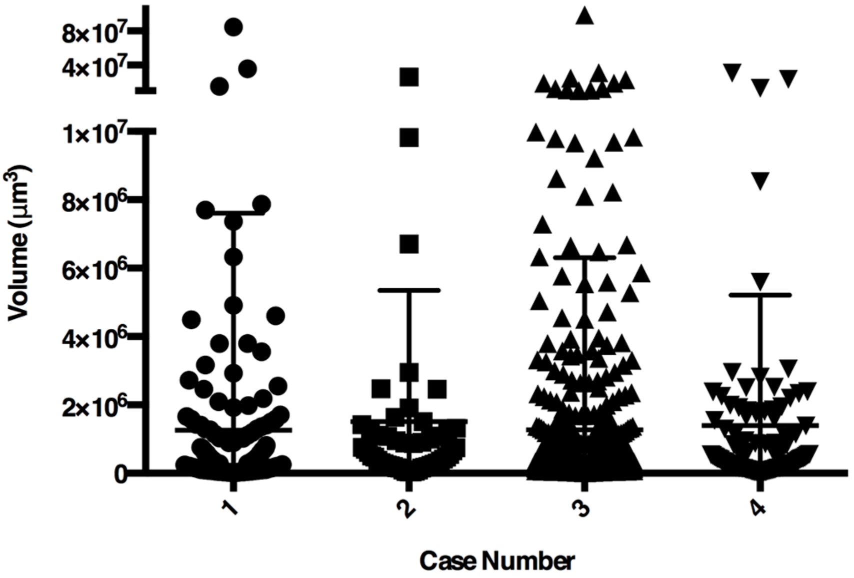

Figure S3. Review of co-aligned Movat's pentachrome-stained sections of an area of usual interstitial pneumonia tissue demonstrates one morphologically simple fibroblast focus in 3D. Illustrative photomicrographs. In (D) the focus is identified by the black arrow. In (I) the focus is no longer visible. Section depth in microns is indicated for each photomicrograph. Scale bar in (A) is 200 µm.

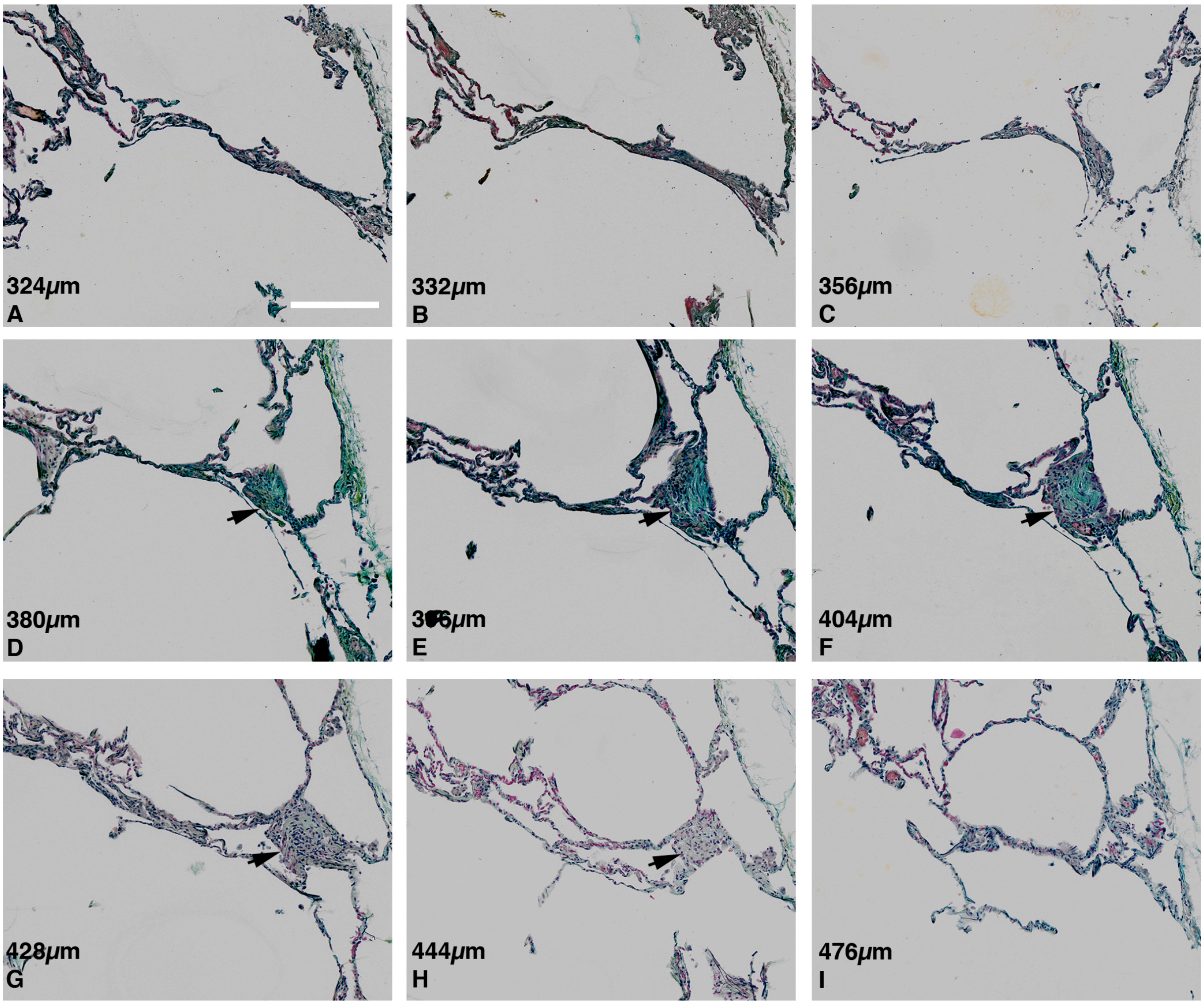

Figure S4. Quantitative and semi-quantitative analyses of fibroblast focus profusion within tissue samples. Fibroblast focus profusion every 20–30 μm over approximately 1000 μm was measured on tissue sections semi-quantitatively (FF score, scale 0 – 6) or quantitatively (fibroblast foci per cm^2 of tissue) from each tissue sample (n=4). (A) Case 1, (B) Case 2, (C) Case 3, (D) Case 4.

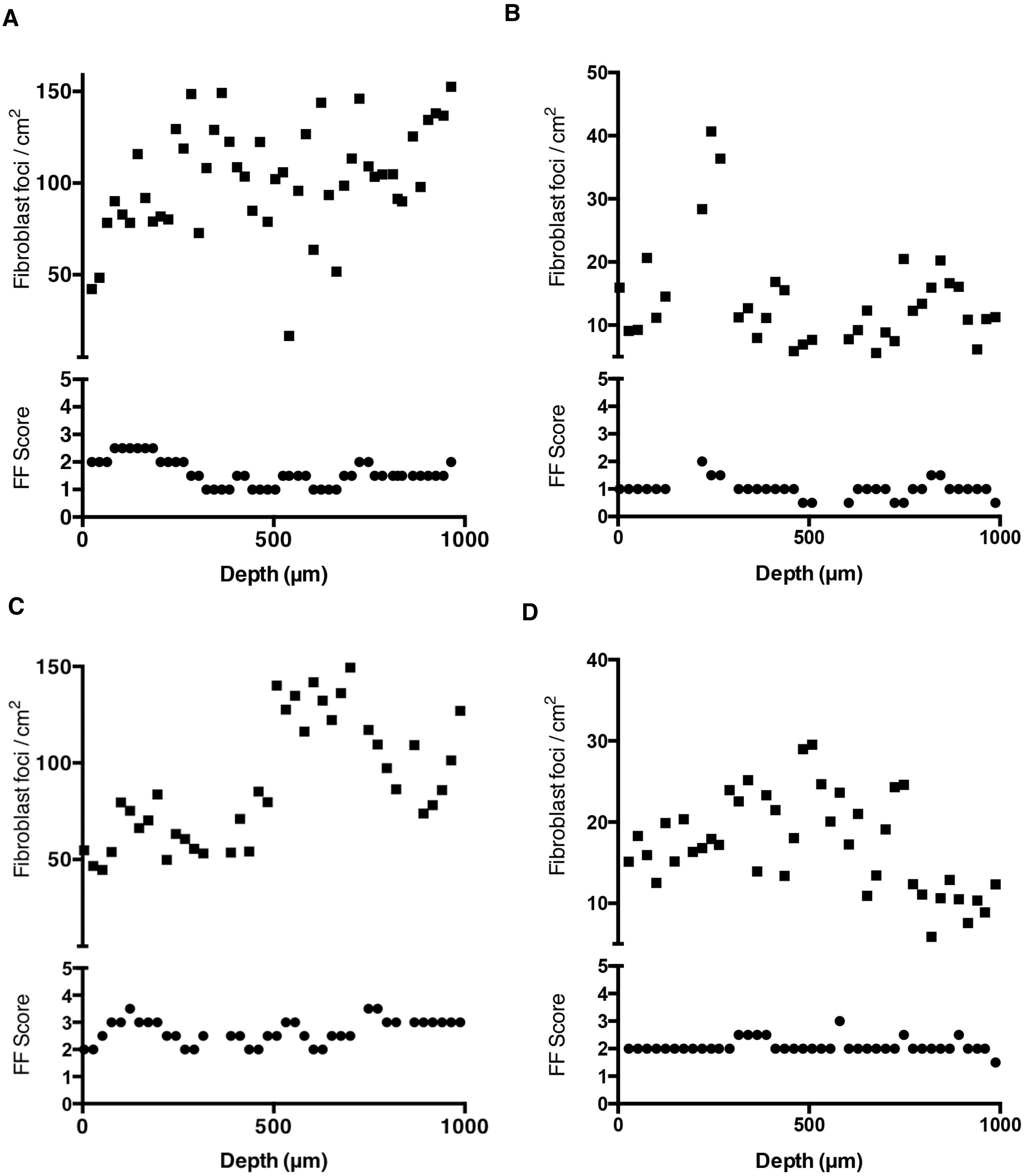

Figure S5. Correlation of fibroblast focus density (foci per mm^3 of lung tissue) with one year forced vital capacity (FVC) percentage change as assessed by linear regression (P=0.04; R^2=0.92)

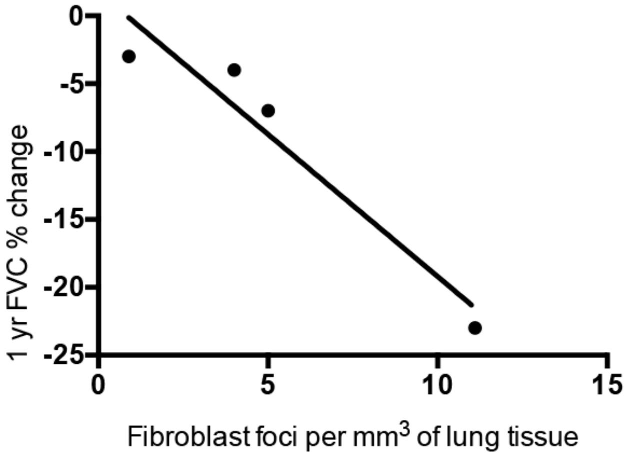

Figure S6. Thresholding to remove paraffin from the microCT volume. (A) An area of lung tissue with paraffin present. The mean intensity of paraffin is calculated from a 1 mm^3 volume of paraffin for lung tissue segmentation based on absolute thresholding shown in (B). This allows subtraction of the background of paraffin from the microCT volume as displayed in (C). In (D) a 3D volume rendering of lung tissue prior to paraffin removal is presented. In (E) the corresponding volume following paraffin removal is shown. Scale bar in (A) is 2 mm.

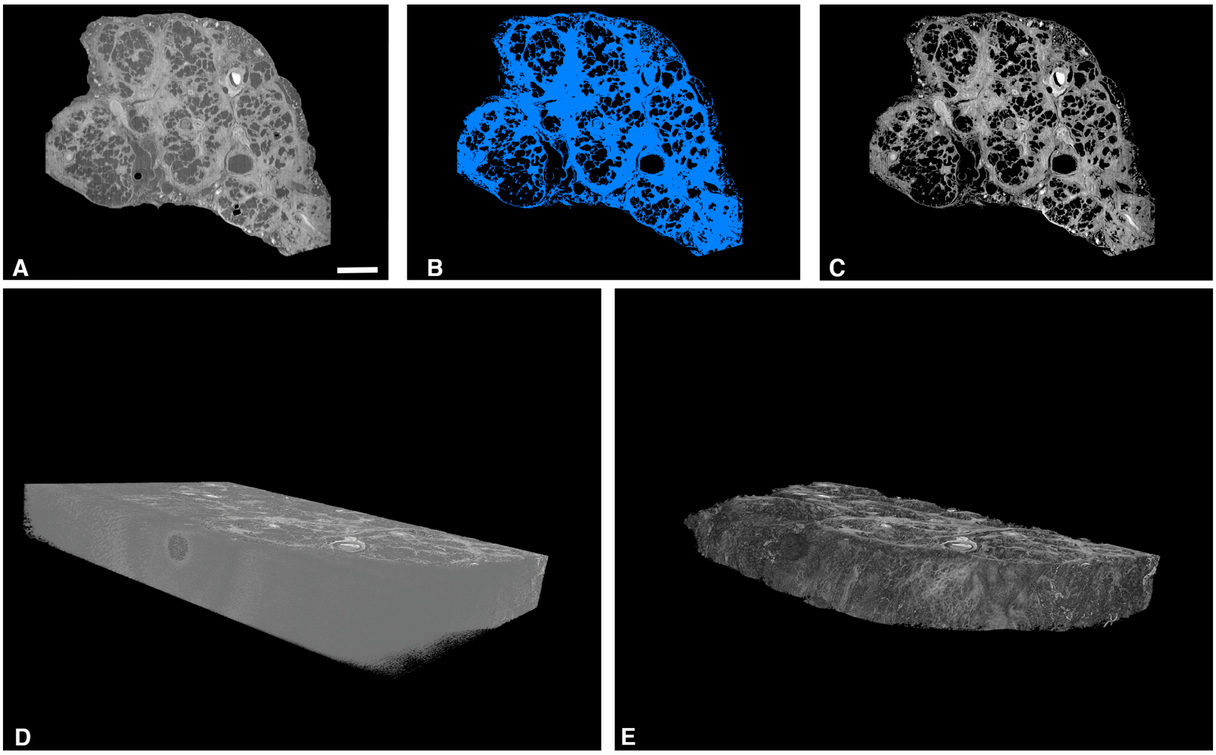

Supplementary Video Legends

Video 1. Multi-planar and 3D visualisation of IPF lung tissue imaged by microCT. IPF lung tissue is visualised in multiple planes, and a lung tissue volume visualised in 3D with and then without paraffin, demonstrating that microCT of paraffin embedded tissue can visualise the microscopic lung structure of fibrotic lung diseases.

Video 2. 3D volume rendering of normal lung tissue imaged by microCT. A cube (2 mm per side) of normal lung tissue was extracted from the reconstructed microCT lung tissue volume. For reference on the superior surface a co-registered histological section stained with Movat's Pentachrome-stain is displayed at the end of the video.

Video 3. 3D volume rendering of UIP/IPF lung tissue imaged by microCT. A cube (2 mm per side) of UIP/IPF lung tissue was extracted from the reconstructed microCT lung tissue volume. For reference on the superior surface a co-registered histological section stained with Movat's Pentachrome stain is displayed at the end of the video.

Video 4. 3D visualisation of one fibroblast focus. As serial 2D microCT images are stripped, the 3D reconstruction visualises the complex morphology of one fibroblast focus (blue). For reference, at the end of the video the pleural surface is displayed (yellow)

Video 5. 3D visualisation of multiple fibroblast foci. Multiple fibroblast foci are visualised in this 3D reconstruction, demonstrating they form discrete structures with wide variations in morphology. For reference, the IPF lung tissue is visualised in multiple planes, and a volume of lung tissue is visualised in 3D.

Supplementary Render Legend

Render 1. A 3D model in the STereoLithography (STL) format of one fibroblast focus following fibroblast focus segmentation and volume rendering within AvizoFire 7·1 (FEI, Oregon, USA). Voxel size is 8 μm in all dimensions.

Supplementary References

S1. Ochs M, et al. The Number of Alveoli in the Human Lung. *Am J Respir Crit Care Med.* 2004;169(1):120–124.

S2. Gundersen HJ, et al. The New Stereological Tools - Disector, Fractionator, Nucleator and Point Sampled Intercepts and Their Use in Pathological Research and Diagnosis. *APMIS.* 1988;96(10):857–881.

S3. Cool CD, et al. Fibroblast Foci Are Not Discrete Sites of Lung Injury or Repair: The Fibroblast Reticulum. *Am J Respir Crit Care Med.* 2006;174(6):654–658.

S4. Nyengaard JR, Marcussen N. The number of glomerular capillaries estimated by an unbiased and efficient stereological method. *J Microsc.* 1993;171(1):27–37.

S5. Wulfsohn D, Knust J, Ochs M, Nyengaard JR, Gundersen HJG. Stereological estimation of the total number of ventilatory units in mice lungs. *J Microsc.* 2010;238(1):75–89.

S6. Gundersen HJ, Boyce RW, Nyengaard JR, Odgaard A. The Conneulor: unbiased estimation of connectivity using physical disectors under projection. *Bone.* 1993;14(3):217–222.

S7. Nicholson AG et al. The relationship between individual histologic features and disease progression in idiopathic pulmonary fibrosis. *Am J Respir Crit Care Med.* 2002;166(2):173–177.
